# Association between combination antibiotic therapy as opposed as monotherapy and outcomes of ICU patients with *Pseudomonas aeruginosa* ventilator-associated pneumonia: an ancillary study of the iDIAPASON trial

**DOI:** 10.1186/s13054-023-04457-y

**Published:** 2023-05-30

**Authors:** Arnaud Foucrier, Thomas Dessalle, Sophie Tuffet, Laura Federici, Claire Dahyot‑Fizelier, François Barbier, Julien Pottecher, Antoine Monsel, Tarik Hissem, Jean‑Yves Lefrant, Alexandre Demoule, Jean‑Michel Constantin, Alexandra Rousseau, Tabassome Simon, Marc Leone, Adrien Bouglé, iDIAPASON Trial Investigators, Adrien Bouglé, Julien Amour, Thomas Dessalle, Florence Bellenfant Zegdi, Bernard Cholley, Julien Massot, Jean-Michel Constantin, Alexandre Demoule, Julien Mayaux, Vincent Dubée, Hervé Dupont, Jacques Duranteau, Laura Federici, Arnaud Foucrier, Thomas Geeraerts, Céline Guichon, Pierre Kalfon, Éric Kipnis, Sigismond Lasocki, Jean-Yves Lefrant, Matthieu Legrand, Marc Leone, Thomas Lescot, Bruno Lévy, Joël Cousson, Philippe Montravers, Sébastien Tanaka, Emmanuel Novy, Alexandre Ouattara, Jean-François Payen, Walter Picard, Pascale Poète, Julien Pottecher, Christophe Quesnel, Muriel Fartoukh, Anoine Tesniere, Mélanie Fromentin, Jean-Jacques Rouby, Qin Lu, Olivier Langeron, Pierre Squara, Eric Levesque, Nicola Mongardon

**Affiliations:** 1 Department of Anesthesiology and Critical Care, Beaujon Hospital, DMU Parabol, AP-HP Nord, Université de Paris, Clichy, France; 2 Department of Anesthesia, Critical Care and Perioperative Care, Pitié-Salpetrière Hospital, 47-83, Boulevard de l’Hôpital, 75013 Paris, France; 3 Department of Clinical Pharmacology-Clinical Research Platform, AP-HP, Sorbonne University, Paris, France; 4 Service de Réanimation Polyvalente, Centre Hospitalier d′Ajaccio, Ajaccio, France; 5 Department of Anaesthesia and Intensive Care, University Hospital of Poitiers, Poitiers, France; 6 Service de Médecine Intensive-Réanimation, Centre Hospitalier Régional d’Orléans, 14, Avenue de l’Hôpital, 45100 Orléans, France; 7 Hôpitaux Universitaires de Strasbourg, Hôpital de Hautepierre, Department of Anaesthesiology, Critical Care and Perioperative Medicine, Fédération de Médecine Translationnelle de Strasbourg, ER 3072, Strasbourg University Hospital, Strasbourg, France; 8 Multidisciplinary Intensive Care Unit, Department of Anesthesiology and Critical Care, GRC 29, AP-HP, Pitié-Salpêtrière Hospital, Sorbonne University, Paris, France; 9 General Intensive Care Unit, Sud-Essonne Hospital, Étampes, France; 10 UR-UM103 IMAGINE, Univ. Montpellier, Division of Anesthesia Critical Care, Pain and Emergency Medicine, Nîmes University Hospital, Montpellier, France, Nîmes University Hospital, Montpellier, France; 11 Service de Médecine Intensive et Réanimation (Département R3S), APHP, Site Pitié-Salpêtrière, Sorbonne Université, Paris, France; 12 Service d’anesthésie et de Réanimation, Hôpital Nord, Assistance Publique Hôpitaux de Marseille, Aix Marseille Université, Marseille, France; 13 Department of Anesthesiology and Critical Care Medicine, Cardiology Institute, GRC 29, AP-HP, Pitié-Salpêtrière Hospital, Sorbonne University, 47-83 Boulevard de l’Hôpital, 75013 Paris, France

**Keywords:** Ventilator-associated pneumonia, *Pseudomonas aeruginosa*, Antibiotic therapy, Combination therapy

## Abstract

**Background:**

The optimal treatment duration and the nature of regimen of antibiotics (monotherapy or combination therapy) for *Pseudomonas aeruginosa* ventilator‑associated pneumonia (PA-VAP) remain debated. The aim of this study was to evaluate whether a combination antibiotic therapy is superior to a monotherapy in patients with PA-VAP in terms of reduction in recurrence and death, based on the 186 patients included in the iDIAPASON trial, a multicenter, randomized controlled trial comparing 8 versus 15 days of antibiotic therapy for PA-VAP.

**Methods:**

Patients with PA-VAP randomized in the iDIAPASON trial (short-duration—8 days vs. long-duration—15 days) and who received appropriate antibiotic therapy were eligible in the present study. The main objective is to compare mortality at day 90 according to the antibiotic therapy received by the patient: monotherapy versus combination therapy. The primary outcome was the mortality rate at day 90. The primary outcome was compared between groups using a Chi-square test. Time from appropriate antibiotic therapy to death in ICU or to censure at day 90 was represented using Kaplan–Meier survival curves and compared between groups using a Log-rank test.

**Results:**

A total of 169 patients were included in the analysis. The median duration of appropriate antibiotic therapy was 14 days. At day 90, among 37 patients (21.9%) who died, 17 received monotherapy and 20 received a combination therapy (*P* = 0.180). Monotherapy and combination antibiotic therapy were similar for the recurrence rate of VAP, the number of extra pulmonary infections, or the acquisition of multidrug-resistant (MDR) bacteria during the ICU stay. Patients in combination therapy were exposed to mechanical ventilation for 28 ± 12 days, as compared with 23 ± 11 days for those receiving monotherapy (*P* = 0.0243). Results remain similar after adjustment for randomization arm of iDIAPASON trial and SOFA score at ICU admission.

**Conclusions:**

Except longer durations of antibiotic therapy and mechanical ventilation, potentially related to increased difficulty in achieving clinical cure, the patients in the combination therapy group had similar outcomes to those in the monotherapy group.

*Trial registration*: NCT02634411, Registered 15 December 2015.

**Supplementary Information:**

The online version contains supplementary material available at 10.1186/s13054-023-04457-y.

## Background

Ventilator-associated pneumonia (VAP) is one of the most frequent ICU-acquired complications. VAP affects between 5 and 40% of mechanically ventilated patients [1] and is associated with prolonged durations of mechanical ventilation, of ICU length of stay and an attributable mortality from 1 to 10% [2, 3]. *Pseudomonas aeruginosa* (PA) is one of the most common bacteria causing VAP [4]. PA-VAP is associated with a crude mortality of 42.1–87% and a high attributable mortality of 32.0–42.8%, even among patients receiving appropriate antimicrobial therapy [5, 6]. Incidence of PA-VAP treatment failure (PA-VAP recurrence or death) is as high as 36%, even in patients receiving a combination antibiotic therapy [7]. In two retrospective studies, the use of monotherapy or combination therapy in the definitive regimen did not affect the rates of mortality or recurrence [8, 9]. The US guidelines suggest combination therapy for patients with PA-VAP with septic shock or those at high risk of death [10], whereas the French guidelines suggest preferring monotherapy in this setting [11]. Despite these recommendations, a cluster-randomized trial reported that de-escalation of antibiotic therapy was achieved in less than half of patients managed for hospital-acquired pneumonia [12]. The randomized controlled trial iDIAPASON was conducted to evaluate optimal duration of antibiotic treatment in PA-VAP. Non-inferiority of 8-day group compared to the 15-day group was not demonstrated for the primary outcome combining mortality and PA-VAP recurrence occurring during the ICU stay until day 90 (difference 9.7%, 90% CI − 2.4 to 21.9%), probably due to a high number of recurrences in the 8-day group [13]. PA-VAP remains associated with high mortality and recurrence rates, and few studies are available to describe the management of antibiotic therapy in these patients. We therefore aimed to investigate in a well-defined cohort of prospectively included patients whether the use of monotherapy or combination therapy in the definitive antibiotic regimen was associated with an increased risk of mortality, PA-VAP recurrence, and emergence of resistance.

## Methods

The iDIAPASON trial [13] was a prospective, multicenter, randomized controlled trial (NCT02634411) designed to compare a composite endpoint combining mortality and PA-VAP recurrence occurring during the ICU stay until day 90, according to an 8-day or 15-day duration of antibiotic therapy in intensive care unit (ICU) patients with PA-VAP, confirmed by quantitative culture of a respiratory sample. A total of 196 patients (88 assigned to receive 8 days and 98 to receive 15 days of antibiotic therapy) were enrolled in 30 centers between June 2016 and May 2018. Study protocol and ethical aspects are detailed elsewhere [13, 14].

Adult patients were eligible in iDIAPASON trial if they met the following criteria for PA-VAP: a clinical suspicion (≥ two criteria including fever > 38·5 °C, leukocytosis > 10^9^/L or leukopenia < 4.10^8^/L, purulent tracheobronchial secretions, and a new or persistent infiltrate on chest radiography) and confirmation by a *P. aeruginosa* positive quantitative culture of a respiratory sample: broncho-alveolar lavage fluid (significant threshold ≥ 10^4^ colony-forming units (CFU)/mL) or plugged telescopic catheter (significant threshold ≥ 10^3^ CFU/mL) or quantitative endotracheal aspirate pulmonary secretion samples (significant threshold ≥ 10^6^ CFU/mL), according to international definition. Patients were not eligible in case of the following conditions: pregnancy, immunosuppression (HIV, immunosuppressive therapy, corticosteroids > 0.5 mg/kg per day for more than a month), current antibiotic therapy active on *P. aeruginosa* for extra-pulmonary infection, procedure of withdrawing life-sustaining treatment, chronic pulmonary colonization with *P. aeruginosa* (chronic obstructive pulmonary disease (COPD) or bronchiectasis, with a positive respiratory sample below the threshold rate for *P. aeruginosa* (i.e., < 10^3^ CFU/mL for protected specimen brush, < 10^4^ CFU/mL for broncho-alveolar lavage or < 10^6^ CFU/mL for tracheal aspirate), obtained in the absence of pneumonia or exacerbation during the 6 months before the ICU admission).

Antibiotic therapy was initiated after bacteriological respiratory sampling, without waiting for the results of microbiological analysis (bacteria identification and/or results of antimicrobial susceptibility testing—AST). The choice of initial antibiotic therapy was left to the discretion of the physician according to usual care based on the clinical context, previous antibiotic therapy, the presence or absence of risk factors for multidrug-resistant (MDR) pathogen or hospitalization in the previous 90 days (current hospitalization ≥ 5 days, mechanical ventilation ≥ 5 days, support in a dialysis center or residency in a nursing home), local epidemiological data, and finally, knowledge that the patient is already known as being colonized by a MDR pathogen. In the presence of risk factor of MDR pathogen and/or in case of septic shock, broad-spectrum antibiotic was recommended immediately, with the association of a β-lactam/β-lactamase inhibitor or an antipseudomonal cephalosporin, and an aminoglycoside or an antipseudomonal fluoroquinolone for 3–5 days. Initial antibiotic therapy with a narrow spectrum was possible in case of early-onset pneumonia (mechanical ventilation < 5 days) and in the absence of risk factors for MDR pathogens. In all cases, investigators were strongly encouraged to adapt the initial regimen into a narrower spectrum therapy, based on culture results and AST. Antibiotic therapy had to be interrupted, either at the end of day 8 or day 15, according to the randomization group, excluding antibiotic therapy for a documented pulmonary infection recurrence or a new extra-pulmonary infection before that day.

The primary outcome of the present study was the mortality rate at day 90. The secondary outcomes included PA-VAP recurrence rate, invasive mechanical ventilation duration, ICU stay duration, antibiotic exposure duration, number and types of extra pulmonary infections, and acquisition of MDR pathogens (from swab sample of rectum and anterior nares).

Recurrence was defined with a post hoc diagnosis by two independent experts blinded to the treatment arms with predefined criteria: clinical suspicion of VAP after at least 48 h without active antibiotic therapy for *P. aeruginosa*, defined as the association of at least of one of the following signs (fever > 38.5 °C, leukocytosis > 10^9^/L or leukopenia < 4.10^8^/L) with purulent tracheobronchial secretions and a new or persistent infiltrate on chest radiography, then confirmed with a positive quantitative culture, as described above. In cases of disagreement between the two experts (C.-E. L., F.B.), a third expert (C. D.-F.) will reach a consensus.

### Definitions

- Empirical therapy was considered as treatment (monotherapy or combination therapy) that was initiated when the VAP was suspected and before reception of AST.
- Appropriate empirical antimicrobial therapy was defined if empirical therapy included at least one antibiotic active against the isolated strain. Isolates with intermediate levels of susceptibility were classified as resistant.
- Combination therapy consisted of the combination of a β-lactam and an aminoglycoside or a fluoroquinolone or an association of a fluoroquinolone and an aminoglycoside.
- Definitive antibiotic therapy was defined as antibiotic regimen after the clinicians had adapted it according to the AST results.
- Definitive monotherapy was defined as only one active antimicrobial (a β-lactam, fluoroquinolone, or colistin), and considered as appropriate antibiotic therapy, administered all along duration of treatment
- Definitive combination therapy included a β-lactam and an aminoglycoside or a fluoroquinolone or an association of fluoroquinolone and an aminoglycoside, and considered as appropriate antibiotic therapy and administered all along duration of treatment.

### Statistical analysis

Baseline characteristics were reported according to the type of definitive antibiotic therapy (“definitive monotherapy”/“definitive combined therapy”), using frequencies and percentages for categorical data and using mean and standard deviation (sd) or median and interquartile range (IQR) for continuous data, according to their distribution and differences between groups were tested using Chi-squared or Fisher Exact test and Student t-test or Wilcoxon–Mann–Whitney test, respectively.

The proportion of patients with definitive antimicrobial treatment based on combination of antibiotics was compared between “15-day” and the “8-day” groups of the iDIAPASON trial using a Chi-square test.

The primary outcome was compared between groups using a Chi-square test. Time from appropriate antibiotic therapy to death in ICU or to censure at day 90 was represented using Kaplan–Meier survival curves and compared between groups using a Log-rank test. The proportion of patients with recurrent VAP in the ICU and the proportion of patients with MDR pathogens acquired during the ICU stay were compared between groups using a Chi-square test. The duration of mechanical ventilation and ICU length of stay were compared between groups using a Wilcoxon–Mann–Whitney test. The number of extra pulmonary infections was compared between groups using a Wilcoxon–Mann–Whitney test. The type of extra pulmonary infections was described.

Sensitivity analyses were performed for all outcomes using model adjusted for randomization arm of the iDIAPASON study and SOFA score at inclusion. Logistic regression models were used for binary outcome, linear regression models for quantitative outcome after log-transformation, Cox proportional hazard model for survival time outcome and generalized linear model with negative-binomial distribution for count data (number of extra pulmonary infections). Considering ICU mortality and recurrence of VAP event, supplementary sensitivity analysis was made using propensity score (PS) approach. PS was built modeling the type of antibiotherapy using logistic regression model including age, gender, randomization arm of the original study, BMI and respiratory, cardiovascular, renal, neurological and hematological SOFA item as covariates. Hepatic SOFA item and the presence of at least one comorbidity at the admission were not included in the model, considering that they were associated with the type of antibiotic therapy administered (monotherapy or combination) but not with mortality nor recurrence of PA-VAP. The effect of type of antibiotic therapy on mortality on the one hand and on recurrence of PA-PAVM, on the other hand, was estimated using logistic regression models stratified on the quintiles of PS.

All analyses were performed with the SAS software version 9·4 statistical software (SAS Institute Inc. Cary, NC, USA). All tests were two-sided and a P-value of less than 0.05 indicated statistical significance. No adjustment for multiple comparisons was made.

## Results

A total of 169 patients were included in the present study among the 186 analyzable patients in iDIAPASON, after exclusion of 14 patients with inappropriate empirical antimicrobial therapy and 3 patients who did not receive any empirical antimicrobial treatment. Empirical treatment was based on combined antibiotics in 113 (66.9%) patients. Definitive antimicrobial treatment was based on combined antibiotics in 75 (44.4%) patients without difference between the “15-day” and the “8-day” groups of the iDIAPASON trial (*P* = 0.641).

**Fig. 1:**
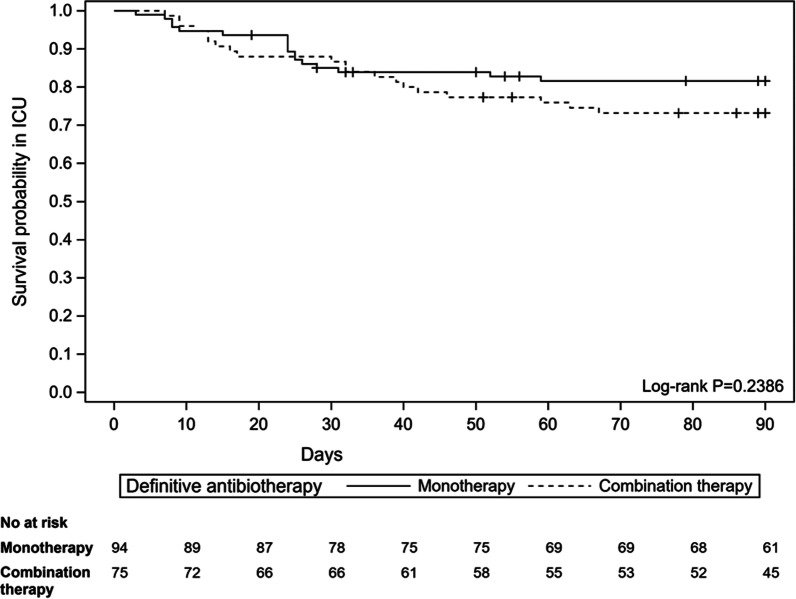
Survival curves of the survival probability for death in ICU (Kaplan–Meier estimates). Survival probability is for the 90 days since the start of appropriate antibiotic therapy

Characteristics of the study population are presented in Table 1. The different antibiotic regimens are detailed in the additional files (Additional file 1: Table S1 and Additional file 2: Table S2). The median [IQR] duration of appropriate antibiotic therapy was 14.0 days [8;15] in the study population, 10.5 days [8;15] in the monotherapy group versus 15.0 days [9;16] in the combination antibiotic therapy group (*P* = 0.0006). Thirty-seven patients (21.9%) died during hospitalization in ICU until Day 90, 17 of whom received monotherapy and 20 of whom received a combination therapy (*P* = 0.1801). Overall survival in ICU until Day 90 was similar in both groups in unadjusted and adjusted analysis (HR 1.48 (0.77–2.82), *P* = 0.2386**,** Fig. 1)**.** There was no difference between monotherapy and combination antibiotic therapy for recurrence of VAP (*P* = 0.319), the number of extra-pulmonary infections in the ICU (*P* = 0.897), or the acquisition of MDR (*P* = 0.737) during ICU stay (Table 2). These results were maintained after adjustment for the randomization arm of the original study and SOFA score at inclusion and after stratification on PS quintiles for ICU mortality and recurrence of VAP (Additional file 3: Table S3).

**Table 1:** Baseline characteristics of studied population

	Monotherapy *N* = 94	Combination therapy *N* = 75	*P*-value
Baseline characteristics			
Sex (male)	73 (77.7)	54 (72.0)	0.3976
Age (years)	61.9 ± 16.7	57.7 ± 17.1	0.1106
Main diagnosis			0.7108
Cardiovascular pathology	21 (22.3)	19 (25.3)	
Trauma	15 (16.0)	14 (18.7)	
Acute respiratory failure	19 (20.2)	15 (20.0)	
Sepsis	14 (14.9)	7 (9.3)	
Postoperative	11 (11.7)	8 (10.7)	
Neurological disorder	7 (7.4)	3 (4.0)	
Acute kidney injury	0 (0)	1 (1.3)	
Hemorrhagic shock	4 (4.3)	2 (2.7)	
Metabolic impairment	1 (1.1)	1 (1.3)	
Burn	0 (0)	3 (4.0)	
Category of admission			0.8999
Scheduled surgery	21 (22.3)	19 (25.3)	
Medical	40 (42.6)	31 (41.3)	
Urgent surgery	33 (35.1)	25 (33.3)	
Main comorbidities			0.0487
Heart failure	10 (10.6)	14 (18.7)	0.1374
Diabetes mellitus	12 (12.8)	5 (6.7)	0.1903
Arterial hypertension	27 (28.7)	16 (21.3)	0.2731
Patients with COPD	9 (9.6)	5 (6.7)	0.4956
BMI (kg/m^2^)^a^	27.7 ± 6.7	26.2 ± 6.0	0.1195
Clinical characteristics at inclusion			
Temperature (°C)^b^	36.8 ± 1.3	36.6 ± 1.3	0.1777
Heart rate	95.6 ± 25.7	98.6 ± 24.0	0.4372
Systolic arterial pressure (mmHg)	125.5 ± 32.9	122.5 ± 27.9	0.5305
Diastolic arterial pressure (mmHg)	65.4 ± 17.8	70.8 ± 17.2	0.0467
Mean arterial pressure (mmHg)	85.4 ± 19.6	88.0 ± 18.6	0.3880
Catecholamines (µg/kg/min)			0.1129
No	43 (45.7)	28 (37.3)	
Dobutamine ≤ 5µg/kg/mn	0 (0)	2 (2.7)	
Dobutamine > 5 µg/kg/mn or norepinephrine ≤ 0,1µg/kg/mn	8 (8.5)	13 (17.3)	
Dobutamine > 15µg/kg/mn or norepinephrine > 0,1µg/kg/mn	43 (45.7)	32 (42.7)	
PaO_2_/FiO_2_^c^	244.5 [157.0; 367.5]	218.0 [126.0; 330.0]	0.2306
Leucocytes (G/L)^d^	13.3 [9.1; 20.2]	13.5 [8.0; 19.8]	0.5250
Procalcitonin (µg/L)^e^	0.9 [0.3; 8.8]	3.8 [0.4; 12.6]	0.3300
Glasgow score (points)^f^	13.0 [3.0; 15.0]	15.0 [7.0; 15.0]	0.1483
SOFA score (points)	7.4 ± 4.3	7.7 ± 3.4	0.6340

Data are expressed in n (%) or mean ± sd or median [inter-quartile range]

Missing values:

^a^8 in monotherapy group

^b^1 in combination therapy group

^c^10 in each group

^d^2 in monotherapy group

^e^74 in monotherapy group and 59 in combination therapy group

^f^6 in monotherapy group and 2 in combination therapy group

**Table 2:** Summary of the results of the comparative analyses between adapted monotherapy and combination therapy

	Monotherapy *N* = 94	Combination therapy *N* = 75	*P*-value
ICU mortality	17 (18.1)	20 (26.7)	0.1801
Recurrence of VAP	15 (16.0)	8 (10.7)	0.3190
Number of days under mechanical ventilation^a^	23.0 [12.0; 34.0]	28.0 [16.5; 50.0]	0.0243
Length of stay in intensive care unit (days)	33.0 [21.0; 51.0]	38.0 [25.0; 60.0]	0.0654
Number of extra pulmonary infections during ICU stay^b^	1.0 [0.0; 2.0]	0.0 [0.0; 2.0]	0.8971
MDR pathogens acquired during ICU stay^c^	18 (19.8)	16 (21.9)	0.7372

Data are expressed in n (%) or median [inter-quartile range]

Missing data values

^a^3 in each group,

^b,c^3 in monotherapy group and 2 in combination therapy group

**Fig. 2:**
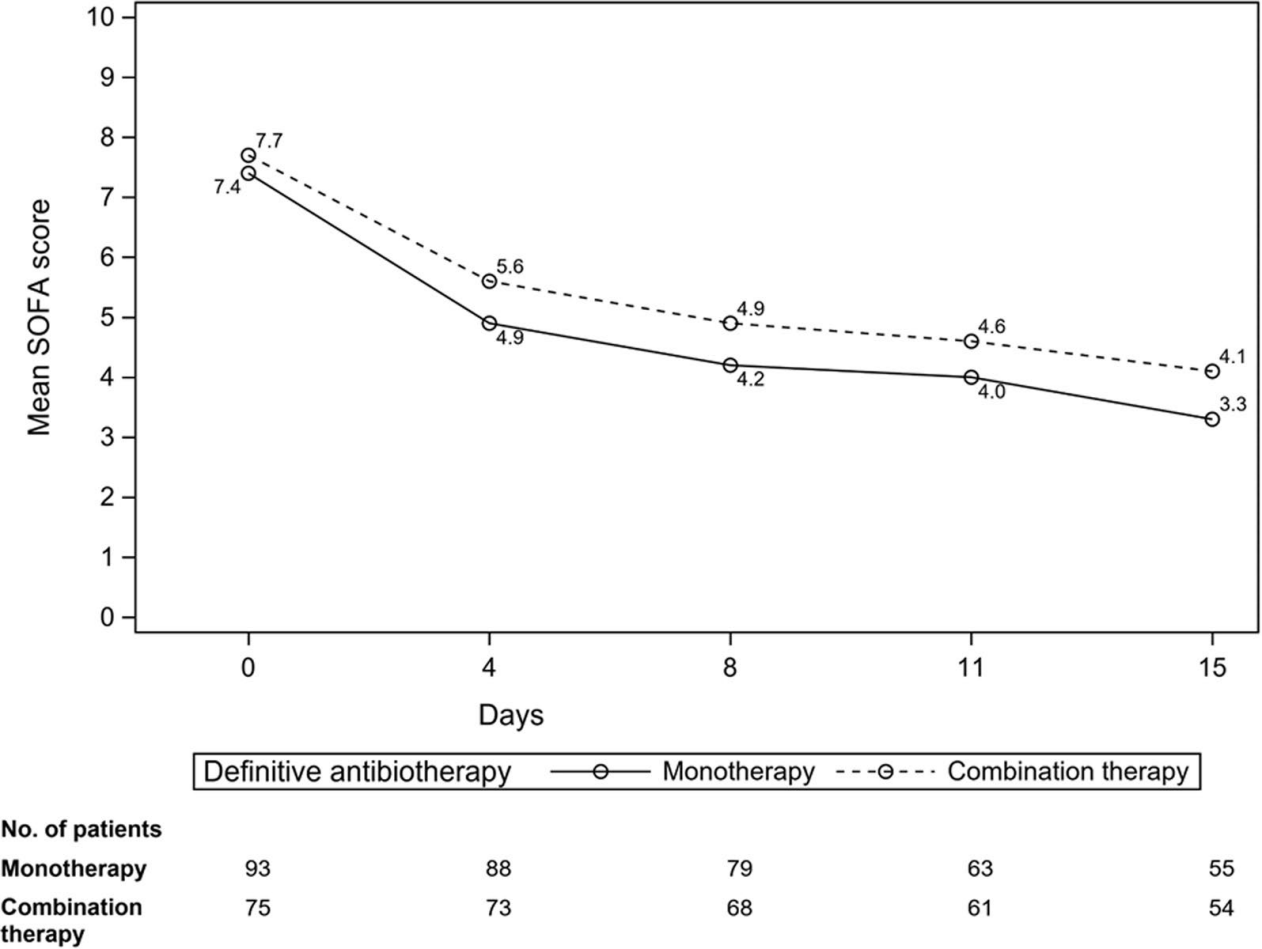
Evolution of the SOFA score according to the type of adapted antibiotic therapy

The median duration of mechanical ventilation for the combination therapy group and the monotherapy group was 28 days [16.5; 50.0] days and 23 [12.0; 34.0] days, respectively (*P* = 0.024) (Table 2). This result was maintained after adjustment in a linear regression model (ß (95% CI) 0.27 (0.03–0.51), *P* = 0.028) (Additional file 3: Table S3). The rate of antibiotic changes due to resistance profile alteration of PA or persisting PA despite treatment was 28.4% in the combination therapy group and 5.4% in the monotherapy group (*P* < 0.001). During treatment, the evolution of the SOFA score was similar between the two groups (Fig. 2). The ICU length of stay in patients who received a combination of antibiotics for definitive antibiotic therapy was 38 [25.0; 60.0] days for combination therapy, compared with 33 [21.0; 51.0] days for monotherapy (*P* = 0.065) (Table 2).

## Discussion

In this study nested in a prospective randomized controlled trial on the duration of antibiotic therapy for PA-VAP, we did not show any difference between a combination of antibiotics and a monotherapy for definitive antibiotic therapy in terms of mortality rate at day 90, recurrence of VAP, number of extra-pulmonary infections or acquisition of MDR pathogen during ICU stay. In contrast, the patients who received a combination of antibiotics had a longer duration of mechanical ventilation. Although optimal antibiotic management for the treatment of VAP remains an issue, only few randomized controlled trials have addressed such results for PA-VAP.

Based on most expert opinion, a combination of antibiotics is recommended to manage infections due to *P. aeruginosa*, especially in the case of severe infections [8, 9, 15].

This recommendation is based on in vitro data [16] and old studies including immunosuppressed patients. This recommendation was confirmed in a cohort of 136 patients treated for extensively drug-resistant *P. aeruginosa* pneumonia. The survival analysis showed an association between survival and combination antibiotic therapy over appropriate monotherapy [17].

Both the IDSA and European guidelines [18] recommend the use of empirical dual antibiotic therapy against *P. aeruginosa* in high-risk patients (septic shock and/or risk of antimicrobial resistance). For definitive treatment, the international guidelines are in favor of maintaining dual antibiotic therapy only in the most severe patients, whereas the European guidelines consider that dual antibiotic therapy is probably necessary only in cases of infection caused by an XDR or PDR pathogen.

In contrast, our findings suggest that there was no advantage to use a combination therapy in terms of outcome, including MDR emergence. These results are consistent with available data in the literature. In a retrospective, multicenter, observational, cohort study, Garnacho-Montero et al. [8] showed that initial use of combination therapy significantly reduced the likelihood of inappropriate therapy, which was associated with higher risk of death. However, administration of only one appropriate antimicrobial or combination therapy provided similar outcomes, suggesting that switching to monotherapy once the susceptibility is documented was feasible and safe. Similarly, in the 100 patients with PA-VAP included in a retrospective cohort, initial combination therapy increased the likelihood of appropriate therapy but did not seem to affect mortality [9].

The rate of VAP recurrence identified in our study was 13.6%. This rate was intermediate between the study of [19, 20] (27 and 26.8%, respectively) and that of Garnacho-Montero et al. (8.8 and 5% in the definitive single or combined antibiotic therapy groups, respectively). However, we did not show evidence of difference in the rate of VAP recurrence between the single and combined antibiotic therapy groups (16 and 10.7%, respectively, *P* = 0.3). Regarding de-escalation strategy, Leone et al. [21] reported that a de-escalation strategy in bacterial infections was associated with an increase in super infections, although this result was not found in the respiratory infections subgroup. Our study supports the latter data as the occurrence of extra-pulmonary infections was similar in both strategies adopted, definitive single or combined therapy.

Importantly, our results did not suggest an increased risk of acquisition of resistance due to the use of monotherapy, as it supported by two meta-analysis [22, 23]. Despite a similar clinical progression between the two groups, duration of mechanical ventilation (MV) was longer in the combination group. This result is more probably related to the challenge of treating certain subgroups of patients than a direct correlation between the “combination therapy” strategy and the increase in the duration of MV. In our study, a quarter of the patients were treated for more than 15 days, suggesting a pejorative evolution of the *P. aeruginosa* phenotype under treatment, secondary to the acquisition of resistances to the antibiotics initially administered. This hypothesis is supported by a higher rate of patients with change in treatment due to persistence and/or mutation of *P. aeruginosa* in the combination therapy group, thus potentially explaining the prolonged duration of mechanical ventilation.

### Limits

According to the protocol of the iDIAPASON trial [14], therapeutic de-escalation was recommended as soon as AST of *P. aeruginosa* was identified. In combination therapy group, the increase in antibiotic duration, mechanical ventilation duration, persisting *P. aeruginosa* and its higher mutation rate during treatment suggests that, according to current recommendations, the patients included in this group corresponded in part to those with worse outcomes.

## Conclusion

In this ancillary study of the iDIAPASON trial, the use of combination therapy versus monotherapy was not associated with a difference in mortality or PA-VAP recurrence in ICU at day 90. An increase in mechanical ventilation duration was observed in the combination group.

### Supplementary Information

**Additional file 1. Table S1**: Regimen of antibiotic therapy.

**Additional file 2. Table S2**: Summary of the results of the comparative analyses between adapted monotherapy and combination therapy.

**Additional file 3. Table S3**: Results of the comparative analyses between monotherapy and combination therapy adjusted for the randomization arm of iDIAPASON study and SOFA score. 

## Data Availability

The datasets analyzed during this study are included and are available from the corresponding author on reasonable request.

## Abbreviations

AST: Antimicrobial susceptibility testing
CFU: Colony forming units
ICU: Intensive care unit
MDR: Multidrug resistant
PA-VAP: *Pseudomonas aeruginosa* ventilator-associated pneumonia
PDR: Pan-drug-resistant
VAP: Ventilator-associated pneumonia
XDR: Extensively drug-resistant
