# Supplementary material for: Association between combination antibiotic therapy as opposed as monotherapy and outcomes of ICU patients with Pseudomonas aeruginosa ventilator-associated pneumonia: an ancillary study of the iDIAPASON trial
Source: Crit Care. 2023 May 30;27:211. doi: 10.1186/s13054-023-04457-y (PMC10230680; doi:10.1186/s13054-023-04457-y)
Supplement: Supplementary file 1 — Additional file 1. Table S1: Regimen of antibiotic therapy. [file 13054_2023_4457_MOESM1_ESM.docx]

| **Empirical antibiotic therapy** |  |
| --- | --- |
| Effective empirical antimicrobial therapy, N (%) | 169 (90) |
| **Type of effective empirical antibiotic therapy**  Appropriate agent when combination* |  |
| ß-Lactam | 46 (27.2) |
| Aminoglycoside | 8 (4.7) |
| Fluoroquinolone | 1 (0.6) |
| Colistin | 1 (0.6) |
| Effective combination^#^ |  |
| ß-Lactam + aminoglycoside | 101 (59.8) |
| ß-Lactam + fluoroquinolone | 7 (4.1) |
| ß-Lactam + aminoglycoside + fluoroquinolone | 1 (0.6) |
| Fluoroquinolone + aminoglycoside  > 2 antibiotics | 2 (1.2)  2 (1.2) |
| **Type of definitive antibiotic therapy**  **Definitive monotherapy**  ß-Lactam  Colistin  **Definitive combination therapy**  ß-Lactam + aminoglycoside  ß-Lactam + fluoroquinolone  Fluoroquinolone + aminoglycoside  ß-Lactam + cotrimoxazole  > 2 antibiotics | **94 (55.6)**  92 (54.3)  2 (1.2)  **75 (44.4)**  39 (23.1)  16 (9.5)  3 (1.8)  2 (1.2)  15 (8.9) |
| delay of antibiotherapy adjustment (days) | 3 |
| switch combination to monotherapy | 62 (36.6) |
| **Total duration of antibiotic therapy** (days) |  |
| overall | 14 [8;15] |
| combination therapy | 15 [9;16] |
| monotherapy | 10 [8;15] |

**Table S1. Regimen of antibiotic therapy**

Data are expressed as n (%) or median [inter-quartile range]

*if association and only the mentioned antibiotic is active

^#^ all antibiotics are active
