# Supplementary material for: Association between combination antibiotic therapy as opposed as monotherapy and outcomes of ICU patients with Pseudomonas aeruginosa ventilator-associated pneumonia: an ancillary study of the iDIAPASON trial
Source: Crit Care. 2023 May 30;27:211. doi: 10.1186/s13054-023-04457-y (PMC10230680; doi:10.1186/s13054-023-04457-y)
Supplement: Supplementary file 2 — Additional file 2. Table S2: Summary of the results of the comparative analyses between adapted monotherapy and combination therapy. [file 13054_2023_4457_MOESM2_ESM.docx]

**Table S2**. Exposition of antibiotics

| **Variable** | **monotherapy N=94** | | **Combination therapy N=75** | | **P-value** | |  |
| --- | --- | --- | --- | --- | --- | --- | --- |
| **Total Duration of antibiotic exposition in ICU (days)** |  | 19.0 [13.0 ; 29.0] |  | 23.0 [15.0 ; 36.0] | | 0.0571 | |
| **Duration of effective antibiotic therapy (days)** |  | 10.5 [8.0 ; 15.0] |  | 15.0 [9.0 ; 16.0] | | 0.0006 | |

Data are expressed as median [inter-quartile range]
