# Supplementary material for: Association between combination antibiotic therapy as opposed as monotherapy and outcomes of ICU patients with Pseudomonas aeruginosa ventilator-associated pneumonia: an ancillary study of the iDIAPASON trial
Source: Crit Care. 2023 May 30;27:211. doi: 10.1186/s13054-023-04457-y (PMC10230680; doi:10.1186/s13054-023-04457-y)
Supplement: Supplementary file 3 — Additional file 3. Table S3: Results of the comparative analyses between monotherapy and combination therapy adjusted for the randomization arm of iDIAPASON study and SOFA score. [file 13054_2023_4457_MOESM3_ESM.docx]

**Table S3.** Results of the comparative analyses between monotherapy and combination therapy adjusted for randomization arm of iDIAPASON study and SOFA score at inclusion or of propensity score stratified analysis.

|  |  | **Adjusted model *** | | **Propensity Score stratified model** | |
| --- | --- | --- | --- | --- | --- |
| **ICU mortality** |  | **OR (95% CI)** | **P-value** | **OR (95% CI)** | **P-value** |
| Monotherapy |  | 1 |  | 1 |  |
| Combination therapy |  | 1.66 (0.80 ; 3.47) | 0.1745 | 1.66 (0.73 ; 3.76) | 0.2254 |
| **Recurrence of VAP** |  | **OR (95% CI)** | **P-value** | **OR (95% CI)** | **P-value** |
| Monotherapy |  | 1 |  | 1 |  |
| Combination therapy |  | 0.64 (0.25 ; 1.61) | 0.3402 | 0.86 (0.30 ; 2.50) | 0.7801 |
| **Number of days under mechanical ventilation** |  | **Beta (95% CI) ^1^** | **P-value** |  |  |
| Monotherapy |  | 0 |  |  |  |
| Combination therapy |  | 0.27 (0.03 to 0.51) | 0.0276 |  |  |
| **Length of stay in intensive care unit (days)** |  | **Beta (95% CI)** | **P-value** |  |  |
| Monotherapy |  | 0 |  |  |  |
| Combination therapy |  | 0.17 (-0.03 to 0.37) | 0.0891 |  |  |
| **Number of extra pulmonary infections during ICU stay** |  | **RR (95% CI)** | **P-value** |  |  |
| Monotherapy |  | 1 |  |  |  |
| Combination therapy |  | 1.07 (0.65 to 1.78) | 0.7768 |  |  |
| **MDR pathogens acquired during ICU stay** |  | **OR (95% CI)** | **P-value** |  |  |
| Monotherapy |  | 1 |  |  |  |
| Combination therapy |  | 1.13 (0.53 ; 2.41) | 0.7566 |  |  |

* Adjustment for randomization arm of iDIAPASON study and SOFA score at inclusion

^1^ Coefficient estimated after log transformation
